# Rnnotator: an automated *de novo *transcriptome assembly pipeline from stranded RNA-Seq reads

**DOI:** 10.1186/1471-2164-11-663

**Published:** 2010-11-24

**Authors:** Jeffrey Martin, Vincent M Bruno, Zhide Fang, Xiandong Meng, Matthew Blow, Tao Zhang, Gavin Sherlock, Michael Snyder, Zhong Wang

**Affiliations:** 1 Genomics Division, Lawrence Berkeley National Laboratory, Berkeley, California, USA; 2 Department of Energy, Joint Genome Institute, Walnut Creek, California, USA; 3 Department of Molecular, Cellular and Developmental Biology, Yale University, New Haven, CT 06520, USA; 4 School of Public Health, LSU-Health Sciences Center, New Orleans, LA 70112,USA; 5 Department of Genetics, Stanford University Medical School, Stanford, CA 94305-5120, USA

## Abstract

**Background:**

Comprehensive annotation and quantification of transcriptomes are outstanding problems in functional genomics. While high throughput mRNA sequencing (RNA-Seq) has emerged as a powerful tool for addressing these problems, its success is dependent upon the availability and quality of reference genome sequences, thus limiting the organisms to which it can be applied.

**Results:**

Here, we describe Rnnotator, an automated software pipeline that generates transcript models by *de novo *assembly of RNA-Seq data without the need for a reference genome. We have applied the Rnnotator assembly pipeline to two yeast transcriptomes and compared the results to the reference gene catalogs of these organisms. The contigs produced by Rnnotator are highly accurate (95%) and reconstruct full-length genes for the majority of the existing gene models (54.3%). Furthermore, our analyses revealed many novel transcribed regions that are absent from well annotated genomes, suggesting Rnnotator serves as a complementary approach to analysis based on a reference genome for comprehensive transcriptomics.

**Conclusions:**

These results demonstrate that the Rnnotator pipeline is able to reconstruct full-length transcripts in the absence of a complete reference genome.

## Background

RNA-Seq has emerged as a powerful tool for studying transcriptomes. It aims to provide a comprehensive list of all transcripts and their expression levels from a given cell or cell population under a particular condition. A typical RNA-Seq experiment involves RNA isolation followed by conversion to a library of short cDNA fragments and sequencing using next-generation sequencing technology [1,2]. RNA-Seq data analysis typically involves aligning the short read sequences to a reference genome to reveal reads from exons, splicing junctions, or polyA ends. This information is used to i) derive novel gene models or refine existing gene models, including exon structure and untranslated regions (UTRs) and ii) to determine gene expression levels from read count statistics [1,3]. A few software packages have been developed to perform one or more of the above data analysis tasks, including TopHat/Cufflinks [4,5], ERANGE [6] and Scripture [7]. This type of reference-based approach can be very successful if the reference genomes are good quality. However, except for a few model organisms, genome assemblies are often incomplete or unavailable. Similarly, sequencing RNA from complex microbial communities, or metatranscriptome sequencing, also poses considerable challenges for data analysis because the genomes for most of the organisms are not known. Thus, in many cases, reference-based analysis of RNA-Seq data is not possible.

*De novo *assembly of RNA-Seq reads into transcripts has the potential to overcome the above limitations. However, short read assembly itself is very challenging. In general, next-generation sequence data contains large numbers of reads with artifacts originating either from the library preparation step (e.g., PCR) or from the sequencing step (e.g., reads containing errors). These poor quality reads can result in fragmented assemblies or assembly errors. Also, the size of sequencing datasets produced is often very large, and therefore requires substantial memory and long computing times, even for the very efficient De Bruijn graph-based assemblers [8-10]. There are additional challenges specific to assembly of RNA-Seq data. For example, the sequencing coverage among different transcripts can range over five orders of magnitude, depending on transcript abundance and sequencing depth. This causes most short read assemblers to be unsuitable for transcriptome assembly because they assume uniform coverage. Furthermore, a set of standard criteria to evaluate the quality of transcriptome assemblies remains an open question.

To address these challenges, we developed an automated software pipeline, called Rnnotator, for preprocessing of RNA-Seq data followed by reference genome independent *de novo *assembly into transcriptomes. We also developed standards to evaluate transcriptome assemblies that can be generalized to many other transcriptomes. For transcripts with deep sequencing coverage we demonstrate that Rnnotator is capable of producing full-length transcript assemblies. Furthermore, we demonstrate that a *de novo *assembly approach can discover transcripts derived from sequences which are not present in the reference genome.

## Results

### The Rnnotator assembly pipeline

**Figure 1:**
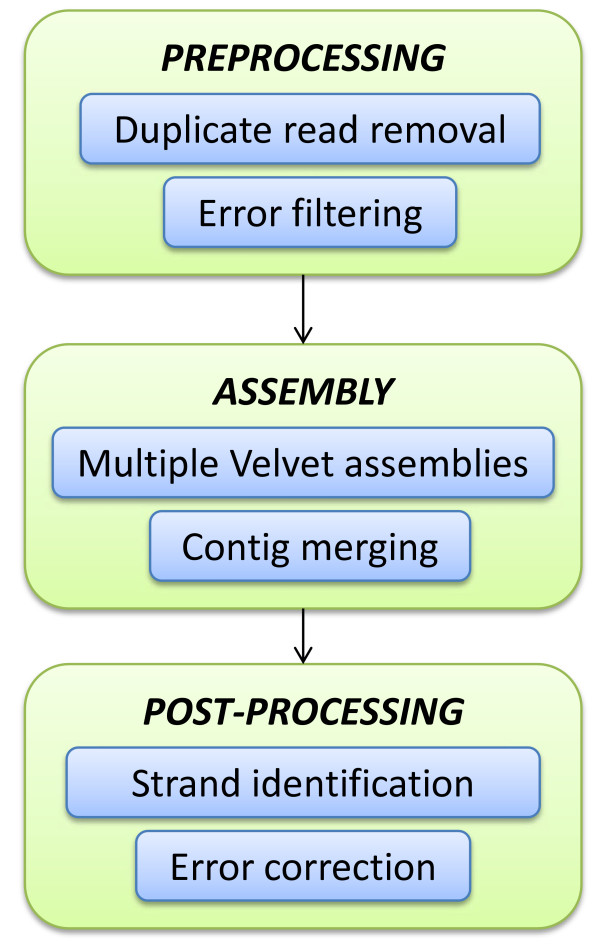
**A summary of the Rnnotator assembly pipeline**.

Rnnotator takes short read sequences as input and outputs assembled transcript contigs. It consists of three major components: preprocessing of reads, assembly, and post-processing of contigs (Figure 1).

**Figure 2:**
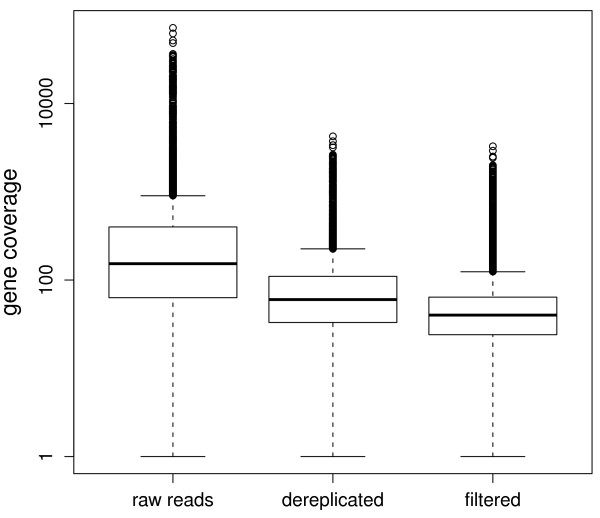
**Read dereplication and filtering greatly reduces the coverage unevenness among genes in RNA-Seq data**. Coverage of reference genes was calculated using raw reads, dereplicated reads, and filtered reads for *Candida albicans *SC5314.

The preprocessing step removes highly redundant reads and low quality sequences found in most RNA-Seq data sets. Large numbers of identical reads may originate from PCR amplification or from abundant transcripts and do not contribute to the assembly. Consolidation of identical reads into a single representative sequence prior to assembly reduces the computational resource requirements for the assembly. Low quality reads containing sequencing errors are also filtered out using a k-mer based approach (Methods). We found that preprocessing the raw reads reduced the variation of gene coverage while improving the computational performance of the assembly significantly. The variance of gene coverage was reduced by 300 fold in *Candida albicans *(Figure 2). These preprocessing steps also reduced the total read count from 186 to 21 million (a reduction of 89%) in the *Candida albicans *SC5314 dataset, which reduced the memory required for one run of Velvet from 46 GB to 5 GB (Table 1).

**Table 1:** Summary of the datasets used in this study

Sequencing Statistics	*C. albicans *(SC5314)	*C. albicans *(WO1)
Number of Lanes	35	26
Read Length	28,34	34
Number of reads	186,148,364	318,539,427
non strand-specific	146,427,272	124,495,811
strand-specific	39,721,092	194,043,616
Unique reads	40,800,738	41,402,683
Median gene coverage of ref. genes	175x	358x

**Figure 3:**
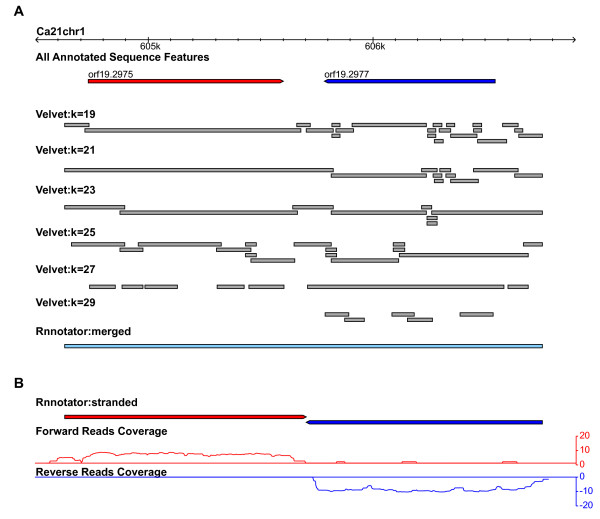
**An example of the assembled transcripts by the Rnnotator pipeline**. **A**) A GBrowse snapshot of assembled transcripts illustrating the effect of different Velvet k-mer parameters. Current annotated genes are shown on top, genes from forward and reverse strand are represented in red and blue, respectively. In grey the assembled contigs for five k-mer lengths are shown. The merged contigs are shown at the bottom. **B**) Contigs are split according to stranded RNA-Seq read coverage (bottom) into transcripts from opposite strands (top). Read coverages are shown in log2 scale, reads originated from the forward strand are shown in red and those from reverse strand are shown in blue.

For assembling the filtered reads Rnnotator uses Velvet [10] as the default assembler. To obtain an optimal set of assembly parameters we tried several different parameter sets and evaluated their performance. Since there is no single parameter set that can give the best results for all genes, we executed multiple Velvet assemblies and then merged the resulting contigs using the Minimus2 assembler from the AMOS package [11]. Merging the Velvet assembled contigs resulted in a much better assembly (an example is shown in Figure 3A).

Rnnotator takes special consideration of the direction of transcription. To determine the transcription direction as well as resolve overlapping transcripts that originate from opposing DNA strands (Figure 3A) Rnnotator incorporates information from strand-specific RNA-Seq reads (Figure 3B, Table 2). It does this by aligning the strand-specific reads to each contig and then splitting contigs at the strandness transition point which signifies the boundary of adjacent transcripts. For genomic regions that have reads from both orientations, indicative of transcript overlap, both strands of the contig are retained after separation (Methods). Finally, single base errors in the assembled contigs are corrected by aligning the reads back to each contig to generate a consensus nucleotide sequence.

**Table 2:** A comparison of the performance between the Rnnotator assembly and a single Velvet assembly.

	Rnnotator (non-stranded)	Rnnotator	Velvet	Oases	Multiple-*k*
*C. albicans *SC5314					
▪ Accuracy^1^	94.0	95.0	97.4	92.3	96.6
▪ Completeness^2^	81.9	80.4	66.7	79.9	85.9
▪ Contiguity^3^	58.4	58.0	46.6	47.9	37.3
▪ Gene fusions^4^	1.73	0.26	1.18	1.31	0.20

***C. albicans *WO1**					

▪ Accuracy	92.8	94.6	96.6	89.1	96.0
▪ Completeness	82.9	82.2	74.0	82.1	88.2
▪ Contiguity	59.1	59.4	43.3	48.6	48.7
▪ Gene fusions	2.06	0.65	1.38	1.61	0.46

^1^Accuracy is defined by the percentage of contigs that share at least 95% identity with the reference genome;

^2^Completeness is the percentage of known genes covered by the contigs to at least 80% of the gene length;

^3^Contiguity is the percentage of complete genes covered by a *single *contig over at least 80% of the gene length.

^4^Gene fusions are the percentage of contigs that contain more than 50% of two or more annotated genes.

### Evaluation of Rnnotator's performance

The ultimate goal of transcriptome assembly from RNA-Seq data is to compile short reads into a set of contigs, each of which represents a full-length transcript, without miss-joining elements of different transcripts or losing the correct representation of the expressed genes. To this end we have developed four criteria: accuracy, completeness, contiguity, and gene fusions to evaluate the quality of the assemblies. Accuracy is a measure of the correctness of the assembly and is estimated by aligning each contig to the reference genome. Completeness measures the degree to which the transcriptome is covered by the assembled contigs and is estimated by calculating the percentage of genes in the annotated gene catalog that are covered at > 80% of the gene length. Contiguity measures the likelihood that a full-length transcript is represented as a single contig and is estimated by calculating the percentage of complete genes covered by a single contig to > 80% of the gene length. Finally, gene fusions measures the number of contigs which contain two genes assembled into a single contig. Using these criteria, we evaluated the performance of Rnnotator against transcriptome assemblies from two strains of a pathogenic yeast species, *Candida albicans *SC5314 and *Candida albicans *WO1 (Table 1).

**Figure 4:**
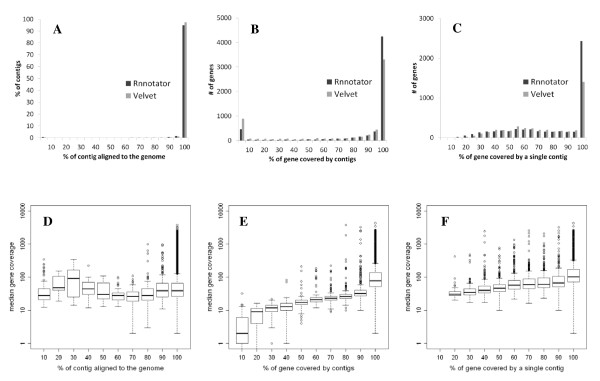
**Accuracy, completeness, and contiguity of assembled transcripts for *Candida albicans *SC5314 are shown in panels (A,D), (B,E), and (C,F), respectively**. For contiguity only genes with > 80% completeness are shown. In panels D), E), and F) a box plot of median gene coverage by unique reads is shown for genes falling into each bin. Open circles above each boxplot depict outliers in the coverage distribution.

To evaluate the accuracy of Rnnotator, we aligned the assembled contigs to the reference genome. For all of the data sets, over 95.0% of the assembled contigs align to the genome at over 95% of the contig length. There is not much difference between the accuracy of Rnnotator and a single Velvet assembly, suggesting that Rnnotator produces highly accurate contigs (Table 2 and Figure 4A and 4D). The accuracy of contigs is not clearly correlated with sequencing depth. Our estimate of accuracy is likely an underestimate of the true accuracy since contigs that represent trans-splicing, which are not straightforward to estimate, are also counted as "misassembled". Rnnotator also determines the orientation for each transcript. This further improves the accuracy, especially in the *Candida *genome where overlapping transcription from opposite strands is very common. For example, from *Candida albicans *SC5314 stranded RNA-seq data, Rnnotator resolved 375 pairs of overlapping transcripts (~10% of the total number of annotated genes).

To evaluate the completeness of the assembly, we compared the Rnnotator assembly with a set of previously annotated genes for each organism. In general, the Rnnotator contigs cover 10-20% more known genes than those from a single Velvet assembly (Table 2); the difference is more pronounced for genes with contigs covering the entire gene length (Figure 4B). As expected, the completeness of the assembly is correlated with the sequencing depth (or expression level) of each gene (Figure 4E). For the ultra-deep sequenced *Candida albicans *SC5314 transcriptome, where the median sequencing coverage of annotated protein coding genes is 175X, 4988 out of 6205 genes (80.4%) have contigs covering at least 80% of their length, demonstrating that Rnnotator is able to produce transcript sequence for the majority of the known yeast genes (Table 2).

We next evaluated the contiguity of the assembly, or how likely a known gene is to be assembled into a single contig covering the full length of the gene. Compared to the results from a single Velvet assembly, Rnnotator assembled many more genes with a single contig covering the entire gene length. In the Rnnotator *Candida *SC5314 assembly 2,893 genes are covered at over > 80% of their length by a single full-length contig, compared to only 1,928 genes from a single Velvet assembly (Figure 4C). Like completeness, contiguity also improves with increasing sequencing coverage (Figure 4F).

We also evaluated the number of contigs containing a gene fusion event. Genes with overlapping UTRs may be joined into a single contig during the assembly process. The Rnnotator contigs exhibited far fewer gene fusion events than the Velvet contigs (Table 2). In the SC5314 assembly, 0.3% of the Rnnotator contigs contained gene fusion events, while 1.2% of the Velvet contigs contain fused genes. Rnnotator is able to drastically reduce the number of fused genes by splitting incorrectly assembled contigs using stranded reads.

In addition to comparing Rnnotator to a single-run of Velvet, we also compared Rnnotator to two other transcriptome assembly strategies: Oases [12] and Multiple-k [13]. For the two Candida data sets tested here, Rnnotator produced contigs with the highest contiguity among the three while its accuracy and completeness are comparable to the other two (Table 2).

These results suggest that full-length transcripts can be accurately *de novo *assembled from ultra-deep RNA-Seq datasets using Rnnotator, and that this tool will be of great value in functional annotation of genes from organisms without sequenced genomes.

### Novel transcribed regions discovered only by de novo assembly

A *de novo *transcriptome assembly has the potential to detect novel transcripts that are not present in the reference genome assembly, or even parasite transcripts that do not originate from the host genome. Of the 18,633 assembled transcripts from the *Candida *SC5314 strain, 150 contigs do not align to the reference genome. However, 97 of these contigs do align to the reference genome of the WO1 strain, suggesting that these contigs are not the result of transcript misassembly or contamination of a foreign species, but instead that the SC5314 genome assembly is incomplete, and/or contains misassemblies. Of the remaining 53 contigs, 23 have BLAST hits to the NCBI non-redundant database (mostly to retrotransposons and hypothetical proteins from *Candida *species). It is possible that these transcripts are derived from the unassembled part of the genome, or they might represent recent genetic additions to the strain used for the experiments. Further experiments are required to resolve these possibilities. The remaining 30 contigs have low complexity sequence and likely originate from sequencing artifacts.

## Discussion

Apart from annotation of the transcriptome, another major goal of RNA-Seq studies is to quantify transcript levels [14]. When a reference transcriptome is available, standard RNA-Seq counting procedures align reads from each sample to the reference gene catalog and the number of reads that align to each gene is used to determine gene expression levels [14]. In the absence of a reference transcriptome, Rnnotator is able to produce a set of transcripts directly from RNA-Seq reads which can serve as the reference, therefore potentially extending the application of gene expression profiling to organisms or metagenome communities that do not have existing transcriptome annotations.

With the sequencing depth used in this study Rnnotator is unable to fully assemble poorly expressed genes that have insufficient sequencing coverage. In cases where there are reference genomes present, this limitation can be partially removed by combining the result from a reference-based transcriptome assembly (such as TopHat followed by Cufflinks [4,5]). While the reference-based assembly will miss transcripts that are derived from unassembled portions of the genome, in the future one would combine these two complementary approaches for a comprehensive annotation of the transcribed regions. Additionally, Rnnotator cannot currently resolve transcripts from duplicated genomic regions, or transcripts produced from polymorphic alleles. A complete re-sequencing of the lab strain used in the manuscript will be required to determine how Rnnotator deals with transcripts from duplicated genomic regions. We assume that near identical transcripts (including those from duplicated regions) will be assembled into one. How transcripts from polymorphic alleles are assembled is also an open question. We assume less abundant alleles will be "corrected" to their abundant counterparts based upon how Rnnotator works. However, allele information should be inferred by mapping raw reads back to the transcripts from those assembled by Rnnotator, a topic that is worth more in-depth exploration. In principle, both of these challenges will be overcome by the increased sequence depth and read length expected from ongoing improvements to DNA sequencing technology.

Finally, it is unknown how alternative splicing will affect transcript assembly. Currently we have not explored transcriptome assembly from an organism in which alternative splicing is prevalent, neither have we had a good reference set that contains a comprehensive list of alternatively spliced transcript variants for evaluation of such effects.

## Conclusion

Here we described a systematic method to assess transcriptome assembly quality by assessing the accuracy, completeness, contiguity, and gene fusion events in transcriptome assemblies. Using these criteria as guidelines, we developed a *de novo *transcriptome assembly pipeline to reconstruct high quality transcripts from short read sequences independent of an existing reference genome, which potentially enables RNA-Seq studies in any organism, simple or complex. We also demonstrated that transcriptome assembly is complementary to reference-based analysis when reference genomes are incomplete. In addition, assembly of RNA-Seq reads also provides an opportunity to discover new types of RNA not encoded in reference genomes.

## Methods

### Library construction and sequencing

The Candida RNA-Seq library construction and sequencing are described elsewhere [15].

### Read quality filtering and duplicate read removal

Condition-specific reads were pooled together and identical reads were removed. After removing duplicate reads, read error filtering was performed using a rare k-mer filtering approach. The frequency of each k-mer was calculated using a hash table and reads containing rare k-mers were not used in the assembly. Rare k-mers were defined as those that occurred less than three times in the set of unique reads.

Several rare k-mer read filtering strategies were tested in order to determine the effect of the read filtering. The three filtering strategies were: i) no filter applied, ii) filter applied after removing duplicate reads, and iii) filter applied before removing duplicate reads (Additional file 1). The order of filtering and duplicate read removal is significant since a k-mer is more likely to be a low abundant k-mer after duplicate read removal than before. We discovered that filtering reads prior to assembly reduces the runtime and memory required by the assembly at the cost of slightly decreasing the assembly quality.

### Multiple Velvet assembly

For assembly of short read Illumina sequences, the Velvet assembler was used in conjunction with the AMOS assembly package [10,11]. Eight runs of velveth were executed in parallel (once for each hash length, 19 through 33). Next eight runs of velvetg were run in parallel with parameters: cov_cutoff = 1, exp_cov = auto. Prior to merging contigs, all duplicates were removed and contigs were combined into a single FASTA file. The minimus2 pipeline [11], a lightweight assembler which is part of the AMOS package, was run using REFCOUNT = 0 (other parameters default).

### Splitting contigs using stranded RNA-Seq

The protocol used to split misassembled contigs using stranded RNA-Seq reads includes: i) splitting contigs with long stretches of less than three mapped reads which are longer than one read length, ii) orienting contigs in the correct mRNA sense strand orientation, iii) generating a consensus contig by counting the number of A,C,G,T residues at each base position. BWA [16] was used to align the reads to the assembled contigs.

### Aligning contigs to the reference

The UCSC Blat software [17] was used to align contigs to both genome and transcriptome references. For yeast datasets the maximum intron size was set to 5,000. In all cases, only the best hits were taken, unless there were multiple best-scoring hits. The score of each alignment was calculated by the formula: *s *= matches - mismatches, as recommended. A similar strategy was used when aligning gene models to contigs (SC5314), again only taking the best scoring hits.

### Detecting gene fusion events

Gene fusion events were detected by first aligning contigs to the reference genome (outlined above). Genomic coordinates for each aligned contig were compared with the genomic coordinates of every annotated gene. A contig and gene were considered overlapping if they shared an overlap which was longer than 50% of the gene length. Contigs containing two or more such genes were identified as containing a gene fusion event.

### Comparing with other assemblers

When performing the single-run Velvet assemblies and the Oases assemblies hash length 21 was used (28 to 34 base pair read lengths). All other parameters were set to the default parameter set. Contigs > = 100 bp in length were used for comparison against other assemblers.

For the Multiple-k assemblies, eight Velvet assemblies were first performed. In order to have a fair comparison against the Rnnotator assemblies, the same hash lengths were used when running Velvet (i.e., 19, 21, 23, 25, 27, 29, 31, 33). The Multiple-k script was then run using the eight Velvet assemblies as input.

### Software Availability

The source code for Rnnotator is available from Lawrence Berkeley National Laboratory under an End-User License Agreement for academic collaborators and under a commercial license for for-profit entities. If you would like to receive this code please contact Virginia de la Puente at vtdelapuente@lbl.gov for details.

## Competing interests

The authors declare that they have no competing interests.

## Authors' contributions

JM, XM and ZW designed and implemented the software. VB and TZ carried out the experiments to generate data. JM, ZF and ZW carried out the analysis. JM, MB, GS, MS and ZW wrote the paper. All authors read and approved the final manuscript.

## Supplementary Material

Additional file 1 **Supplementary Table S1**. Effect of k-mer filtering on assembly quality. Comparisons were performed using the SC5314 dataset. Click here for file
