# Supplementary material for: Rnnotator: an automated de novo transcriptome assembly pipeline from stranded RNA-Seq reads
Source: BMC Genomics. 2010 Nov 24;11:663. doi: 10.1186/1471-2164-11-663 (PMC3152782; doi:10.1186/1471-2164-11-663)
Supplement: Additional file 1 — Supplementary Table S1. Effect of k-mer filtering on assembly quality. Comparisons were performed using the SC5314 dataset. [file 1471-2164-11-663suppl1.pdf]

Supplementary Table 1. Effect of k-mer filtering on assembly quality. Comparisons were performed using the SC5314 dataset.

	**dereplication only**	**dereplication, filter**	**filter, dereplication**
# of reads	40,800,738	21,412,023	19,793,607
Accuracy	95.4	95.0	95.0
Completeness	84.7	80.4	79.3
Contiguity	57.9	58.0	55.9
Runtime (hrs.)	5.5	3.2	5.1
